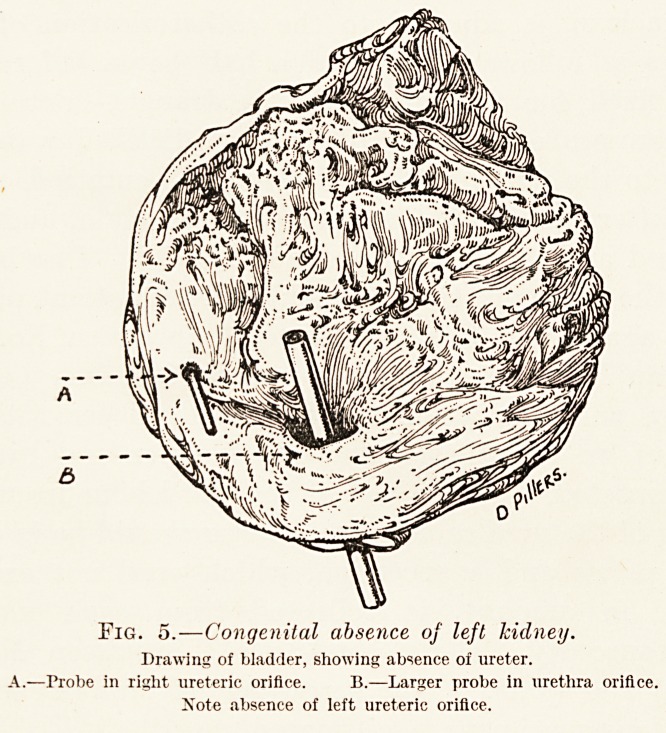# The Investigation of Surgical Cases of Renal Disease
1A postgraduate demonstration.


**Published:** 1926

**Authors:** D. G. C. Tasker

**Affiliations:** Assistant Surgeon, Bristol General Hospital


### PLATE X.

### RENAL DISEASE.

Fig. 1.

Fig. 2.

### THE INVESTIGATION OF SURGICAL CASES OF RENAL DISEASE.[1]

BY

D. G. C. Tasker, B.Sc., M.S., F.R.C.S.,
*Assistant Surgeon, Bristol General Hospital.*

---

So much has appeared lately in the medical literature about renal efficiency from a medical aspect that I thought to-day you would be interested in seeing how modern methods of estimation of renal efficiency are applied to surgery.

I have in the wards a patient, a woman of 48, who for five years has had attacks of typical renal colic, and an X-ray (Fig. 1) shows a large calculus in the right kidney.

I propose to carry out before you to-day, in her case, the examination used as a routine for all renal cases, and I will first briefly explain the steps involved.

Investigation of renal cases falls into four steps :—

1. Clinical examination.
2. X-ray examination.
3. Cystoscopy.
4. Operation.

*The clinical examination* comprises a discussion with the patient of the usual symptoms associated with urinary disease : pain, disturbance of micturition, and the usual clinical examination of the patient abdominally, per rectum, and possibly per urethram, ending with an examination of the urine. Other facts that can be

[1] A postgraduate demonstration.

elicited in the clinical examination of importance in estimating renal efficiency I will mention later.

*X-ray examination* can rarely be omitted, and there are two main points to which I would draw your attention. *First, the whole urinary tract must be X-rayed.* Three films are required in every case, namely, one of each kidney and one of the pelvis to include the bladder and lower portions of the ureters. Even though you are quite confident that the symptoms are due to a lesion in one kidney, the other kidney and the bladder must also be X-rayed. This X-ray (Fig. 2) will emphasise this point. It shows the left renal area of a woman who had typical attacks of renal colic on the left side only. It is entirely negative. At the same time, as a routine, the right renal area was X-rayed

You will see in this X-ray (Fig. 3) several large and very definite stones. Not a pain nor an ache occurred on the right side, yet removal of the stones from the right side cured the pain on the left side. This is not an isolated case, as each year there are usually two or three examples of this referred renal colic found in the X-ray Department of the General Hospital.

Another reason for X-raying the whole urinary tract is that stones in the lower part of the ureter may give rise to pain solely in the loin and renal area, and *vice versa.* Therefore, once you have decided to have a patient X-rayed, insist on three films being taken in every case.

The second point in the X-ray examination is that the *X-rays must be good ones.* This, theoretically, goes without saying ; but a certain amount of time and trouble are required before this is attained, both on the part of the doctor in charge of the case and on the

### PLATE XI.

RENAL DISEASE.

Fig. 3.

Fig. 4.

radiologist's part. Your part consists in the proper preparation of the patient by giving an aperient the night before, and arranging for an enema a few hours before the examination. The enema can be dispensed with, but the aperient never. A practical point is that an oily aperient such as castor oil should not be given, as this seems to leave an opaque coating to the colon making it show up, thus obscuring the renal areas. The preparation by aperient and enema is necessary to get rid of gas and opaque fæcal material in the colon.

The radiologist's part consists of the correct exposure and development of the films, and it is probable that these details are known better by those of you who take their own X-rays than by me. The criteria of satisfactory renal X-rays are :—

1. The colon should be invisible.
2. The outline of the psoas muscle must be clearly visible.
3. The outline of the kidney itself should be seen. This last point is sometimes impossible to attain in fat patients.

However, even with the most careful X-ray examination wrong diagnosis will be made. Fig. 4 shows a collection of small shadows in the right renal area of a man who had had repeated attacks of apparently typical renal colic. These shadows were visible in each of three sets of X-rays taken. Cystoscopy gave no information, and at length I explored the right kidney and ureter, palpating the interior of the kidney and the pelvis with a finger introduced through an incision in the cortex of the kidney. No sign of any stone was found.

The peritoneum was then opened and the biliary apparatus was explored with a negative result. I stitched the right kidney up, as it seemed somewhat mobile, and I am glad to say that the patient has had no symptoms of any kind since. An X-ray taken some six months after the operation shows the shadows still there.

*Cystoscopy* is the next step required in dealing with a renal case. This may seem unnecessary in a patient with a clear shadow showing in the X-ray, but we have to guard against several eventualities that I will detail later.

The cystoscopic examination consists of :—

(1) *Examination of the bladder and ureteric orifices.* This enables cystitis, vesical stone, and papillomata to be excluded. It will also guard one from mistakes in cases having only one kidney and ureter. The ureteric orifices are to the urinary tract what the tongue is to the alimentary canal ; if they are of normal size and not inflamed, it can be taken as certain that there is no gross disease of the kidneys, and *vice versa.*

(2) *Catheterisation of both ureters,* thus enabling a specimen of urine to be withdrawn from both kidneys separately.

(3) *Observation of the rate of excretion of a dye* that has been injected previously into the general circulation.

Thus by cystoscopy we can tell the functional capacity of each kidney separately. It may be that when we come to operate on a kidney in which an X-ray shows a stone, we find that the kidney is hopelessly disorganised ; or (as I have seen happen once) beside a stone being present, there is an entirely unsuspected malignant disease for which nephrectomy is necessary. It then becomes of paramount importance to know whether there is another kidney, and also whether it is sound enough to warrant removal of the kidney in question.

It is, of course, well recognised that a single kidney only may be present. The figure usually given for this is that it occurs about once in every five thousand individuals. Fig. 5 may bring this fact home to you. This is a drawing of the bladder of a patient in which only one kidney was present, and you will see that there is an entire absence of a left ureteric orifice.

FIG. 5.—*Congenital absence of left kidney.*
Drawing of bladder, showing absence of ureter.
A.—Probe in right ureteric orifice. B.—Larger probe in urethra orifice.
Note absence of left ureteric orifice.

Besides the evidence of the presence or absence of a kidney, catheterisation of the ureters tells us whether the other kidney has sufficient functional capacity to allow of removal of the diseased kidney if required; and it also tells us, by the tests I will describe later, whether the capacity of both kidneys together is sufficient to warrant an operation on one kidney. It is no rare occurrence for a patient to have a stone in one kidney, or some condition demanding an operation on the kidney, and for the other kidney to be almost out of action from some latent disease. Patients in this state develop a fatal uræmia with startling suddenness after an operation, however trivial, on one kidney.

To prevent operation on patients with latent uræmia, three tests are carried out :—

1. *Urea Concentration Test.* This test, popularised by Maclean, is adapted to the catheterisation of the ureters as follows : Two and a half hours before the time fixed for the cystoscopy a draught containing 15 grammes of urea in 100 c.c. of distilled water is given to the patient and the bladder is emptied. One hour after this the patient passes urine again, and this is saved and labelled "Specimen No. 1." Two hours after the taking of the urea solution the patient passes urine again, and this constitutes "Specimen No. 2." Half an hour after this the patient is taken to the theatre and the cystoscope is passed, either with or without an anæsthetic, and the bladder and ureters are inspected. By the end of the third hour from the giving of the urea solution a catheter should be in each renal pelvis and a specimen, which need not exceed 5 c.c. in amount, is collected from each kidney simultaneously. These constitute "Specimen No. 3 (right) and (left)." The percentage of urea in each of these four specimens is estimated.

This test consists in giving a large dose of urea and estimating the manner in which the kidney is able to deal with it. If the kidneys have a safe margin of functional capacity they will respond to this excess of urea by excreting it rapidly, so that Specimens Nos. 2 and 3 (right) and (left) will all contain over 2 per cent. of urea. If the percentage of urea is less than 2 per cent. we then know that the kidneys are incapable of dealing with any increased strain put upon them, and that they are very near the threshold of uræmia. Specimen No. 1 is neglected if the result is less than 2 per cent., as it is found that urea in itself is a diuretic and causes an excessive secretion of water which will dilute the percentage of urea in this specimen. This diuresis passes off within an hour, hence the later specimens are unaffected.

By this method of carrying out the urea concentration test (by obtaining the third specimen from each kidney separately) not only can the functional capacity of both kidneys together be estimated but also that of each kidney separately, a most valuable piece of information.

2. *Indigo-Carmine Excretion.* Directly specimens for the preceding test have been obtained, 4 c.c. of a 0·4 per cent. solution of indigo-carmine is injected into a vein in the arm. With a normally acting kidney, within seven minutes and usually at the end of four minutes, puffs of dark blue urine can be seen ejected through each ureteric orifice. If none appears in ten minutes, it is certain that that kidney is of very little functional use. This test will act as a control on the results obtained from the preceding test, and only prolongs the examination by a few minutes.

3. *The estimation of the percentage of urea in the blood* is useful as a control of the capacity of both kidneys together, but it can only be carried out by examination of the blood in a fully-equipped pathological laboratory.

*Clinical Examination.* These tests may seem somewhat complicated, and it may be a relief to you to know that for the purpose of estimating the total renal capacity none of them are more valuable than the evidence that can be obtained from the clinical examination.

The following points in the history give a good notion of the total functional capacity of the kidneys :—

(1) Headaches.

(2) Vomiting, especially unrelated to meals.

(3) Appetite for meat. Loss of appetite is one of the earliest signs of renal impairment.

(4) Thirst.

On examination note should be taken of :—

(5) The tongue ; a dry furred tongue associated with

(6) Dry skin is an almost certain sign of latent uræmia. Other points to be noted are a raised blood-pressure and signs of cardiac enlargement.

The value of these clinical data is of great use when, as sometimes happens, the various chemical tests do not correspond with each other. When there is any doubt the evidence from the clinical examination should be the determining factor.

---

Cystoscopic examination of the patient was then carried out, with the following results :—

Examination of the bladder and ureteric orifices was normal.

Specimen No. 1=2·25 per cent. urea.

Specimen No. 2=2·70 per cent. urea.

Specimen No. 3 (right)=2·80 per cent. urea.

Specimen No. 3 (left)=3·00 per cent. urea.

Indigo-carmine was seen to be excreted through both ureteric orifices in less than seven minutes.

A week later the calculus was removed from the right kidney, and after an uneventful convalescence the patient left the hospital free from symptoms sixteen days after the operation.

## Figures and Tables

**Fig. 1. Fig. 2. f1:**
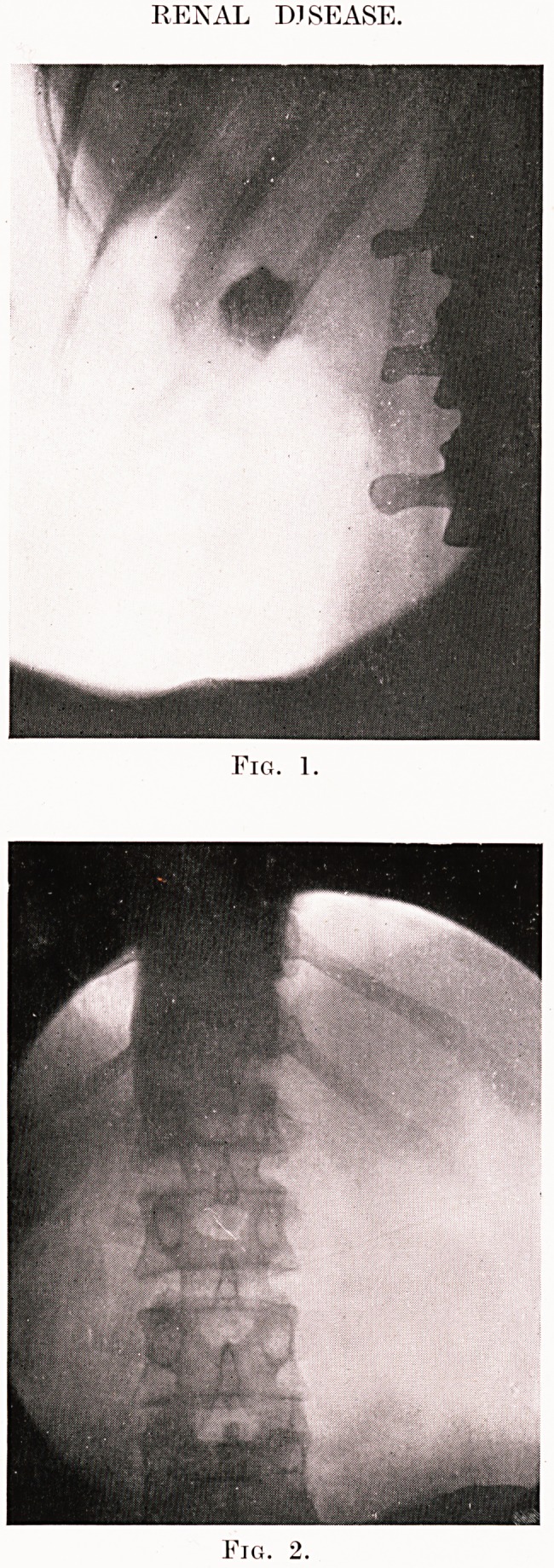


**Fig. 3. Fig. 4. f2:**
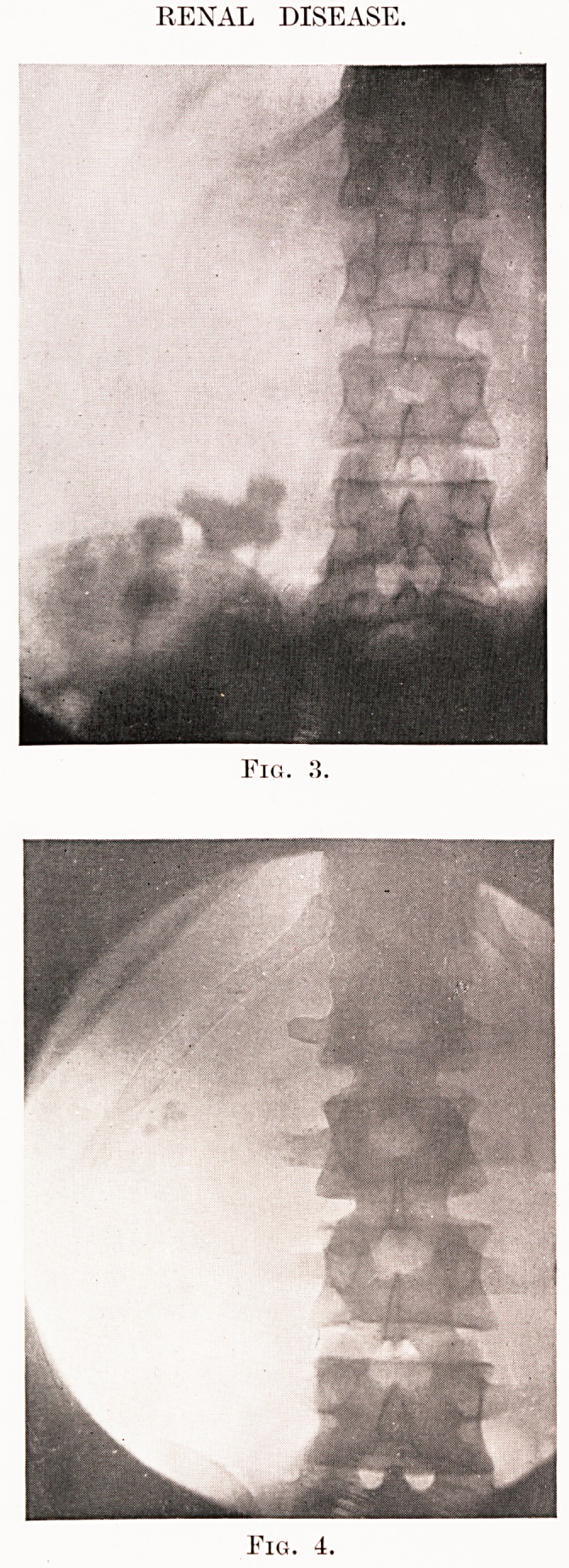


**Fig. 5. f3:**